# Introducing the global medical community to the information presented at local scientific conferences through nephrology blogs

**DOI:** 10.12688/f1000research.1-66.v1

**Published:** 2012-12-14

**Authors:** Tejas Desai, Xiangming Fang, Maria Ferris

**Affiliations:** 1 Division of Nephrology and Hypertension, East Carolina University, Greenville, NC, 27834, USA; 2 Department of Biostatistics, East Carolina University, Greenville, NC, 27834, USA; 3 Division of Nephrology, University of North Carolina – Chapel Hill, Chapel Hill, NC, 27599, USA

## Abstract

An increasing number of healthcare providers author medical blogs (bloggers) to educate the public and fellow physicians. Traditionally, many bloggers have assumed that readers are most interested in information presented at prestigious and popular scientific meetings. As a result, the readers and bloggers often ignore blogs of local scientific meetings. We hypothesize that blog readers will utilize blogs about local scientific meetings less than those about national meetings.

We examined nephrology-pertinent blogs from 2010-2012. Blogs were categorized as "local/regional" or "national/international" based on the majority of the audience that attended the live scientific meeting. We tracked the number of pageviews, reading time, and location of use per blog for the first 90-days after its first availability on the website. Wilcoxon testing was performed on all data.

There were 9 local/regional and 11 national/international scientific meetings for which blogs were available. The mean number of page views was significantly lower in blogs from local/regional than national/international conferences (84.7 versus 160.3, respectively; p < 0.01). However, the mean difference in total reading time between both categories of blogs was not significant (p = 0.25).

Data from this investigation do not fully support the hypothesis that readers utilized local/regional blogs less than national/international blogs. Although local/regional blogs attracted fewer readers (lower pageviews), the content in these blogs was compelling enough to keep the reader equally engaged as with national/international blogs.

## Introduction

An increasing number of healthcare providers author medical blogs (bloggers) to educate the public and fellow physicians ^1– 3^. Bloggers use this medium to report the events, discussions, and controversies that occur at scientific conferences. As a result, the blog is a valuable tool for the reader who may otherwise not have access to this information. Traditionally, many bloggers have assumed that readers are most interested in information presented at prestigious and popular scientific meetings ^4^. Thus, they have focused their blogging efforts on large national and international conferences and have ignored smaller, local meetings ^5^. Historically, local scientific meetings attract a smaller live audience, have a geographically restricted educational impact, and do not present much novel medical information. Nevertheless, the value of blogs that pertain to local conferences has not been studied. Given these limitations, we hypothesize that blog readers will utilize blogs about local scientific meetings less than those about national meetings.

## Methods

We examined nephrology-pertinent blogs authored by the editors or administrators of Nephrology On-Demand ( http://www.mynod.org). These blogs were text-based narrative reports of scientific meetings that occurred between 2010–2012. Blogs were categorized as “local/regional” or “national/international” based on the majority of the audience that primarily attended the live scientific meeting. All of the meetings were based in the United States. The only blogs analyzed were firsthand accounts written by individuals who attended live conferences and not those created from second- or third-party sources. Blogs were posted on Nephrology On-Demand and were freely available to all users at http://goo.gl/28zza. We used Google Analytics to track the number of pageviews, reading time, and location of use per blog for the first 90-days after its availability on the website. Wilcoxon tests were used to compare pageviews and reading time for each blog from different continents. JMP Pro 10 and Microsoft Excel 2007 were used for all statistical analyses.

## Results

**Figure 1.**
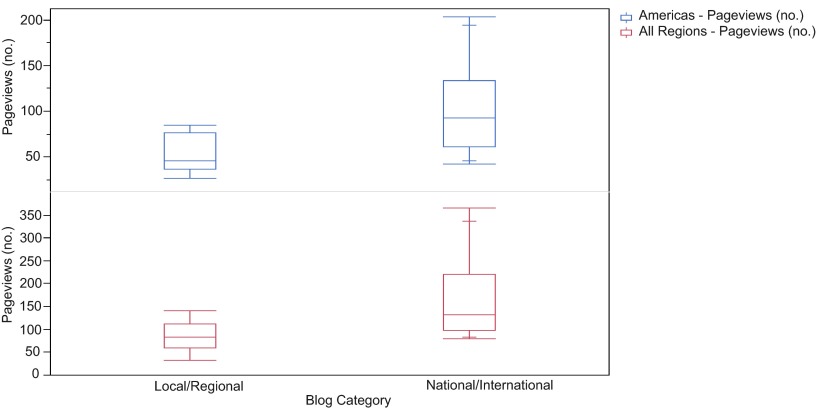
Box Plot of pageviews by blog category. Standard box plot of pageviews of local/regional and national/international nephrology blog posts by readers from all regions (red) and the Americas only (blue) with lines representing minimum value, 25 ^th^ percentile, median, 75 ^th^ percentile, and maximum value within each data set. Where present, inner lines represent 10 ^th^ and 90 ^th^ percentile values.

There were 9 local/regional and 11 national/international scientific meetings for which a blog was available on Nephrology On-Demand ( Table 1). The most popular blogs in each category were “Guest Lecture Series: The Cardiorenal Syndrome” (local/regional; 143 pageviews) and “American Society of Nephrology Renal Week” (national/international; 365 pageviews). Overall, the mean number of pageviews was significantly lower in blogs from local/regional than national/international conferences (84.7 versus 160.3, respectively; p < 0.01) ( Figure 1). For both groups of blogs, the greatest number of pageviews came from the Americas, but there was a significantly lower number of views in local/regional blogs than national/international blogs across all regions ( Table 2).

**Table 1.** Regional category and analysis period of blogs posted on Nephrology On-Demand between 2010–2012.

Blog Category (Type)	Analysis Period	Blog Name	URL*
Local/Regional	7/5/11 - 10/3/11	Guest Lecture Series: Fistula First - Do all comers qualify?	6212
Local/Regional	4/21/11 - 7/26/11	Role of High Blood Pressure in Prevention of CVD	6695
Local/Regional	3/27/11 - 6/25/11	Renal Transplantation Update at East Carolina University	6451
Local/Regional	3/2/11 - 5/31/11	Guest Lecture Series: Physical Exam of AVF	6224
Local/Regional	2/14/11 - 5/15/11	Guest Lecture Series: Glomerular Diseases (in-depth)	5909
Local/Regional	1/24/11 - 4/24/11	Guest Lecture Series: Phosphate binders & Glomerular Diseases	5831
Local/Regional	11/1/10 - 1/30/11	Recent Advances in Internal Medicine at East Carolina University	4944
Local/Regional	3/28/10 - 6/26/2010	Renal Transplantation Update at East Carolina University	3086
Local/Regional	6/6/11 - 9/4/11	Guest Lecture Series: The Cardiorenal Syndrome	5990
National/International	5/18/11 - 8/16/11	American Heart Association’s Epidemiology & CV Disease Meeting	6739
National/International	4/24/11 - 7/29/11	National Kidney Foundation Spring Clinical Meetings	6729
National/International	11/17/10 - 2/15/11	American Society of Nephrology Meeting Renal Week	5161
National/International	9/23/10 - 12/22/10	Peritoneal Dialysis Academy	4748
National/International	9/4/10 - 12/3/10	International Pediatric Nephrology Association Meeting	4564
National/International	7/26/10 - 10/19/10	No. American Dialysis & Transplantation Meeting	4164
National/International	5/3/10 - 8/1/10	American Transplant Congress Meeting	3634
National/International	4/14/10 - 7/13/10	National Kidney Foundation Meeting	3323
National/International	11/11/11 - 2/9/12	American Society of Nephrology Kidney Week	7707
National/International	2/27/12 - 5/27/12	International CRRT Meeting	8057
National/International	5/17/12 - 8/15/12	National Kidney Foundation Spring Clinical Meetings	8289

* URL denotes the prefix: http://blog.ecu.edu/sites/nephrologyondemand/?p.

**Table 2.** Pageviews and reading time by region and blog type (local/regional vs. national/international).

		Pageviews (number)		Time (seconds)	
		Local/Regional	National/International	Local/Regional	National/International
All Regions	Cumulative	762	1764	189407	492973
	Mean	84.7	160	21045	44816
	Std Dev	34.7	83.6	21994	71934
	*p*	*0.0098*		*0.2545*	
Americas	Cumulative	490	1123	131460	333918
	Mean	54.4	102	14607	30356
	Std Dev	21.3	48.9	14752	46586
	*p*	*0.0166*		*0.2545*	
Asia	Cumulative	132	312	28200	112914
	Mean	14.7	28.4	3133	10265
	Std Dev	9.6	20.0	2718	21422
	*p*	*0.0332*		*0.3619*	
Europe	Cumulative	111	217	33082	30186
	Mean	12.3	19.7	3676	2744
	Std Dev	8.8	18.9	5672	2699
	*p*	*0.4243*		*0.4941*	
Oceania	Cumulative	7.0	17.0	1754	3495
	Mean	0.8	1.5	195	318
	Std Dev	0.8	1.9	455	835
	*p*	*0.4485*		*0.5292*	
Africa	Cumulative	19.0	66.0	1449	34321
	Mean	2.1	6.0	161	3120
	Std Dev	2.0	3.3	217	5518
	*p*	*0.0084*		*0.0049*	

**Figure 2.**
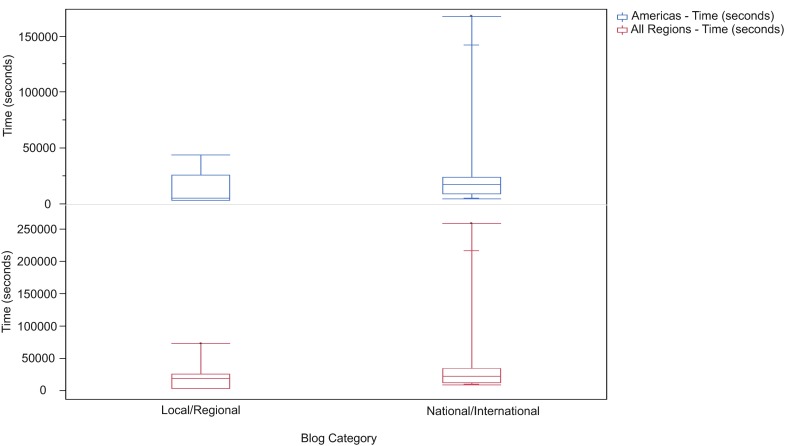
Box Plot of reading time by blog category. Standard box plot of reading time of local/regional and national/international nephrology blog posts by readers from all regions (red) and the Americas only (blue) with lines representing minimum value, 25 ^th^ percentile, median, 75 ^th^ percentile, and maximum value within each data set. Where present, inner lines represent 10 ^th^ and 90 ^th^ percentile values.

Table 2 also indicates the total time spent reading local/regional and national/international blogs. Readers spent a cumulative total of 2.5 times more hours reading national/international than local/regional blogs. However, the mean difference in total reading time between both categories of blogs was not significant (p = 0.25) ( Figure 2). Readers from the Americas spent the greatest amount of total time reading the blogs than from any other region, but there was no statistical difference in the time spent reading either category (p = 0.25).

Nephrology On-Demand 90-day blog metrics Data showing the number of pageviews and reading time of 20 blog posts covering either local/regional or national/international nephrology conferences by readers from different regions. ‘Entrances’ refers to the number of visits to Nephrology On-Demand that occurred through a particular blog; this data was not used in the preparation of the manuscript.

## Discussion and conclusions

Data from this investigation do not fully support the hypothesis that readers utilized local/regional blogs less than national/international blogs. Although local/regional blogs attracted fewer readers (lower pageviews), the content in these blogs was compelling enough to keep the reader equally engaged as with national/international blogs (as there were statistically similar reading times). The latter finding is surprising because it suggests that information presented at local conferences can keep the attention of the reader as effectively as national conferences. Blogs open local conferences to the global community ^6^. In addition, local conferences are conducted at a greater frequency and held at a wider variety of institutions than national/international conferences. The information presented through blogs would be more frequent and present a greater diversity of ideas than blogs of just national/international meetings ^7^.

Further investigations are needed to determine what features local/regional blogs need to have in order to be viewed by a similar number of readers as the national/international blogs. Such features, if identified and incorporated, would greatly increase the value of local/regional scientific conferences. This exploratory investigation suggests that once these readers view a blog, the content within that blog will keep them engaged, no matter where it was presented.
