# Supplementary material for: Introducing the global medical community to the information presented at local scientific conferences through nephrology blogs
Source: F1000Res. 2012 Dec 14;1:66. [Version 1] doi: 10.12688/f1000research.1-66.v1 (PMC3894799; doi:10.12688/f1000research.1-66.v1)
Supplement: Peer review history

# Peer review: Referee response for version 1

**Published:** 2013-01-08

**Authors:** Manish Ponda

**Affiliations:** 1 Laboratory of Biochemical Genetics and Metabolism, The Rockefeller University, New York, NY, USA

I have read this submission. I believe that I have an appropriate level of expertise to confirm that it is of an acceptable scientific standard.

# Peer review: Referee response for version 1

**Published:** 2012-12-20

**Authors:** Tushar Vachharajani

**Affiliations:** 1 Section of Nephrology, W.G. (Bill) Hefner Veteran Affairs Medical Center, Salisbury, North Carolina, USA

A very important and relevant analysis of the use of modern day technology in education. The education tools utilized in medicine in general has to adapt to the needs and interests of the students in the millennium era. I would suggest that the discussion needs to include a few sentences on how best the authors feel the “millennium multi-tasking student” can be kept engaged and focused so the “take home message” is delivered. Will keeping the blogs short and concise help with delivering the right message?

I have read this submission. I believe that I have an appropriate level of expertise to confirm that it is of an acceptable scientific standard.
